# Making Smart Social Judgments Takes Time: Infants' Recruitment of Goal Information When Generating Action Predictions

**DOI:** 10.1371/journal.pone.0098085

**Published:** 2014-05-16

**Authors:** Sheila Krogh-Jespersen, Amanda L. Woodward

**Affiliations:** Department of Psychology, University of Chicago, Chicago, Illinois, United States of America; University of Turin and the Italian Institute of Technology, Italy

## Abstract

Previous research has shown that young infants perceive others' actions as structured by goals. One open question is whether the recruitment of this understanding when predicting others' actions imposes a cognitive challenge for young infants. The current study explored infants' ability to utilize their knowledge of others' goals to rapidly predict future behavior in complex social environments and distinguish goal-directed actions from other kinds of movements. Fifteen-month-olds (N = 40) viewed videos of an actor engaged in either a goal-directed (grasping) or an ambiguous (brushing the back of her hand) action on a Tobii eye-tracker. At test, critical elements of the scene were changed and infants' predictive fixations were examined to determine whether they relied on goal information to anticipate the actor's future behavior. Results revealed that infants reliably generated goal-based visual predictions for the grasping action, but not for the back-of-hand behavior. Moreover, response latencies were longer for goal-based predictions than for location-based predictions, suggesting that goal-based predictions are cognitively taxing. Analyses of areas of interest indicated that heightened attention to the overall scene, as opposed to specific patterns of attention, was the critical indicator of successful judgments regarding an actor's future goal-directed behavior. These findings shed light on the processes that support “smart” social behavior in infants, as it may be a challenge for young infants to use information about others' intentions to inform rapid predictions.

## Introduction

Children's early development is shaped in myriad ways by their interactions with others. Interacting with and learning from others depend on the ability to interpret others' actions as intentional and the ability to deploy this knowledge rapidly in real time to respond appropriately. Converging evidence from several experimental methods has shown that young infants have significant aspects of the first of these abilities, in that they view others' actions as structured by intentions (e.g., [1]–[4]), yet less is known about infants' ability to rapidly recruit their knowledge about others' intentions to predict and respond to actions. The current study investigates whether infants rely on the goal structure of social events when engaging in prospective thinking about others' actions, including a specific examination of whether the goal-directed nature of the action influences their predictions.

Understanding the actions of others requires more than simply attending to agents and their movements; it requires understanding actions as structured by relations between agents and their goals and objects of attention. Infants show sensitivity to the intentional structure of others' actions by 6 to 9 months of age in their responses during visual habituation experiments (e.g., [3]–[6]) and in their tendency to reproduce the goals of others' actions [2], [7]. Even so, this sensitivity is not clearly expressed in overt social behavior at these ages. Between 12 and 24 months of age, infants show dramatic developments in their social interactive abilities. Social interactions require contingent exchanges that are performed with fine-grained temporal precision. The rapid timing required in these contexts may place a cognitive demand on interactive partners beyond the need to analyze one's partner's intentions. The current study examined infants' ability to generate intention-based predictions and assessed the time course in which infants generate these cognitively challenging predictions compared to simpler, movement-based predictions.

Recent eye-tracking studies have shown that infants visually anticipate the endpoints of others' actions, yet leave open the question of whether these anticipatory responses involve an analysis of the actor's intentions or goals. When infants see a hand reaching repeatedly toward an object or location, they look to the object before the hand contacts it (e.g., [8]–[14]). This visual anticipation appears to be faster when there is an object at the final destination (e.g., for actions resulting in containment versus displacement [9]), suggesting that a salient end location is important. Often, results from these action anticipation studies are presented as evidence of infants' understanding of the goal-directness of the actions being performed, yet in these studies, infants can rely on repetition of a completed action and the movement trajectories of the agent's hand as cues to the action's outcome. Therefore, it is not clear from these findings whether infants recruit an analysis of the actor's goal when generating these anticipatory responses because, in these studies, the goal and the trajectory of movement have been confounded. Infants may anticipate the endpoints of repeated, familiar actions in the same way they anticipate other repeated, predictable visual patterns (e.g., [15]) or physical trajectories [16], without considering the goal-directed nature of the action.

To determine whether infants recruit goal information to generate predictions, it is necessary to present test events in which the actions are not completed and the agent's movements are not confounded with the goal of the action. Recently, 11-month-old infants' ability to predict which of two objects a person would reach for based on prior information about the person's goal was measured in a manner that satisfies these requirements [17]. Infants were shown video familiarization events in which a hand reached for and grasped one of two objects. Then, infants viewed a switch trial in which the two objects were now in opposite locations. During the test trial, the objects remained in their new locations, and the hand moved toward the objects but stopped midway between them (i.e., never contacted an object). The question was whether infants would launch a predictive look to one of the objects, and, if so, whether they would predict that the hand would move to the same goal object (now in a new location) or move to the object in the prior location. A second group of infants saw closely matched events in which a mechanical claw, rather than a person's hand, was the actor. Infants launched predictive eye movements to the prior goal object when they viewed a person's hand, and they launched predictive eye movements to the prior location when they viewed the claw. Control analyses indicated that these findings did not derive from differences in infants' attentiveness to the two kinds of events, but rather indicated different patterns of prediction for hand actions as compared to claw movements.

Although these results [17] indicate that infants generate predictions based on the goal structure of an event even in the absence of movement and trajectory cues, other studies aimed at this issue have yielded inconsistent results. One study [18] found that 9-month-old infants relied on frequency or statistical information as opposed to an analysis of the most efficient action when predicting future behavior. Other researchers [19] have demonstrated similar results and further argued that on-line versus retrospective judgments, which are typically tested in passive habituation paradigms, are disassociated from each other until the age of 3 years. Yet, in both of these studies, the stimuli presented were animated nonhuman characters (i.e., a cow and a fish), and infants were given a short time window to process these unusual events and generate a prediction. These results highlight the need for further investigation regarding infants' ability to generate goal-based predictions when viewing rich, animate stimuli, which infants typically encounter in their daily lives, and suggest that an examination of infants' understanding of others' goal-based actions presented within complex environments may provide insight into the time course for generating goal-based predictions.

When engaging in social interactions, individuals have to rapidly recruit and deploy their knowledge regarding their social partner's goals and intentions to support the continuation of the interaction. One possibility is that the rapid timing of these contingent interactions actually imposes a challenge for infants who require larger time windows to generate appropriate responses to their social partners. Indeed, it seems likely that recruiting goal information to generate action predictions involves an additional cognitive burden beyond the prediction of simple movement regularities. As yet, there has not been investigation of the effects of this processing demand on infants' action anticipation. In particular, this additional cognitive effort could be expressed in the speed with which infants generate goal-based versus movement-based predictions, with longer latencies required for the former as compared to the latter. Limitations in infants' ability to use goal information rapidly would have broad implications for the development of social competence. Accordingly, one goal of the current study was to evaluate the time course of infants' goal-based and location-based predictions.

A further goal of the current study was to evaluate how infants' attention to a human agent may relate to their ability to generate fast goal-based predictions. Social environments present a number of information processing demands that could limit the extent to which infants rapidly integrate goal information into their on-line predictions. For one, as infants engage with others in social interactions, they have to update their expectations of the situation as circumstances change. In addition, social partners and their actions are multi-faceted, and so on-line social cognition must contend with information about an actor's face, gaze shifts, and postural movements. These channels of information could support infants' analysis of an actor's goals, but attending to and integrating these channels may also pose an information processing challenge, particularly when time is limited.

In addition, social partners sometimes move in ambiguous ways. Thus, a second challenge is the need to distinguish between goal-directed and unintentional movements, as well as the ability to reason about novel movements that may nonetheless be intentional. Results from visual habituation studies indicate that young infants distinguish between well-formed goal-directed actions, such as grasping, and apparently accidental movements, such as contacting an object with the back of the hand [6], and that infants may be able to use contextual information to interpret a person's ambiguous movements [20]. The current study evaluated whether infants' goal-based action predictions are similarly responsive to differences between well-formed and ambiguous hand actions.

We adapted the paradigm developed by [17] to assess 15-month-old infants' goal predictions when viewing the actions of a woman whose face and upper body were visible. The woman either performed a goal-directed grasping action or an ambiguous action of contacting the object with the back of her hand. Following a single familiarization trial, infants were shown test trials in which the objects' positions were reversed and the woman began her arm movement but paused when her hand was between the two objects. Therefore, during test trials, infants could not rely on movement-regularity or trajectory information when generating action predictions, as the woman never completes the action (i.e., she does not make contact with either object). We assessed whether infants launched predictive eye movements to either the object the woman had previously contacted or the location to which she had previously reached during the familiarization trial. We evaluated the speed with which infants generated these two kinds of predictions in order to test the hypothesis that goal-based predictions are more effortful than location-based predictions. Further, to explore the factors that support “smart” social responses, we evaluated infants' attention to the events prior to generating goal-based and location-based predictions.

## Methods

### Participants

Forty 14- to 16-month-old infants participated in the current study (*M* = 15;01, range: 14;01–16;13 months). Half of the infants were randomly assigned to the Grasp condition (10 males, 10 females; *M* = 14;26) and half to the Back-of-Hand condition (10 males, 10 females; *M* = 15;06). Infants' ages were not significantly different between conditions, *t*(38) = 1.59, *p* = .12. All infants were considered full term (minimum 37 weeks gestation). Participants were recruited from an urban population, and were 40% White, 20% African American, 17.5% Hispanic, 15% Asian and 7.5% multiracial. Given the importance of cumulative gaze information for the data reduction and coding procedures used in the current study, strict criteria for gaze data collection were implemented, leading to an additional 7 infants who were tested and excluded from further analysis due to insufficient data (data collection rate was below 50%) from the Tobii eye-tracker (6) or failure to produce a predictive fixation on either test trial (1).

### Ethics Statement

The Institutional Review Board at the University of Chicago approved the protocol for this study, and written consent was provided by infants' parents/legal guardians prior to participation.

### Procedure

Participants viewed videos presented on a 24-inch monitor equipped with a Tobii T60XL corneal reflection eye-tracking system (accuracy 0.5°, sampling rate 60 Hz). Infants were seated in their parents' laps at an approximate distance of 65-cm from the monitor. Calibration was performed with a 9-point procedure using the standard animation of a bird provided by the Tobii software within the infant calibration setting. When necessary, the calibration process was repeated to improve accuracy. Data were collected and analyzed using Tobii Studio (Tobii Technology, Sweden). The videos had no audio soundtrack.

All infants saw two pre-familiarization trials, one familiarization trial, and two test trials. The pre-familiarization videos for both conditions started with an actor demonstrating that she could reach for a single toy (a novel object) on either side of a table. Next, in a single familiarization trial, she either reached for and grasped (Grasp condition) or touched the back of her hand against (Back-of-Hand condition) one of two objects (a stuffed giraffe or bear). Within each condition, the target object (giraffe vs. bear), the hand the actor used (right vs. left), and the side (right vs. left) on which the target sat were counterbalanced. Half of infants observed a single ipsilateral condition-specific action during the familiarization trial and the other half observed a single contralateral condition-specific action. The hand the actor used to perform the actions was counterbalanced across infants and maintained consistent within infants (i.e., the actor either performed the action with her left or right hand for the familiarization and test trials for an infant). The timing of the actions was controlled in both conditions such that the actor looked at the camera (1-sec), looked down at her hand (.5-sec), raised her hand (1-sec), performed the condition-specific action (2.5-sec), and held the final resting position (2.5-sec). To control for the presence of facial cues during the familiarization trial, the actor looked straight ahead (1-sec), looked down to her hand (.5-sec), watched her hand perform the condition-specific action (2.5-sec), and upon contact with the toy, looked to the contact point where her hand and the toy were conjoined (2.5-sec).

**Figure 1:**
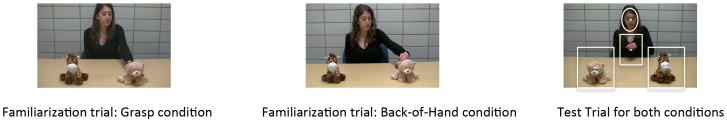
Examples of video stimuli. Depiction of the final video frames for the single familiarization trial in the Grasp and Back-of-Hand conditions, as well as the final video frame for a test trial. AOIs for the person and the objects were identically sized and shaped across conditions for the familiarization trial and for the test trials. The AOIs are depicted here for the test trial image. The individual featured in this figure has given written informed consent (as outlined in PLOS consent form) to publish these images.

Infants in both conditions then viewed the same test videos, with each infant viewing two identical test trials. The objects were shown in reversed locations from their positions in the familiarization trial, and the actor raised her hand and then paused with her hand centered in mid-air between the two objects (see Figure 1). The actor never made contact with either object during the test trials. The timing of the actions in the test trials was as follows: the actor looked at the camera (1-sec), looked down at her hand (.5-sec), raised her hand (1-sec), and held her hand centered between the two objects (5-sec). During the test trials, the actor looked straight ahead (1-sec), shifted her gaze down to her hand as she lifted her hand (.5-sec), and then looked at her hand for the remainder of the test trial. Her hand remained centered between the two objects (5-sec), and she did not look at either object during the test trials.

#### Data reduction

Fixation data were extracted from Tobii Studio to calculate where and when infants fixated during the familiarization and test trials using the data tools available in the program, which include calculating total fixation durations to Areas of Interest (AOIs) and the order in which infants fixated to the relevant AOIs. The AOIs were generated for the actor based on the location of the social information she provided, for example, one AOI encompassed her face and one encompassed the space in which her hand moved during the test trials (i.e., to account for the upward motion).

A total of five static AOIs were created to encompass the female actor's Face and Hand, the Prior Goal and Prior Location objects and the entire viewing screen (see Figure 1). Infants' visual fixations, including their predictive fixations and their attention to the AOIs, were extracted from Tobii Studio. A predictive fixation was defined as a fixation to the actor's Hand AOI followed by a fixation to either the Prior Goal AOI (e.g., the object that the actor acted upon during the familiarization trial) or the Prior Location AOI (e.g., the previously unreferenced object). The AOIs for the objects were located equally distant from the Hand AOI during the test trials. Additionally, the latency (in seconds) for infants to initiate a prediction during each test trial was measured from the start of the test trial to the time that a predictive fixation occurred.

The sizes of the individual AOIs were identical for all video recordings and the AOIs did not differ in spatial relationships across the conditions, allowing for equivalent comparisons of attention. Distribution of infants' attention across the AOIs was calculated using Tobii Studio. The Tobii fixation filter was used to define fixations, which is the default fixation algorithm for Tobii Studio. A fixation was defined as a stable gaze (within 0.75 visual degrees) for a minimum of 200-ms. Saccades through an AOI without a fixation within the AOI were not coded as visual predictions.

## Results and Discussion

The average percentage of fixation data collected did not differ between the Grasp condition (*M* = 76.9%, *SD* = 17.72) and the Back-of-Hand condition (*M* = 71.9%, *SD* = 17.47), *t*(38) = .89, *p* = .38. Three sets of analyses were conducted in order to (1) evaluate whether infants reliably generated goal-based predictions and whether this tendency varied as a function of the action they observed (Grasp versus Back-of-Hand); (2) evaluate the time course for generating goal-based versus location-based predictions; and (3) evaluate the patterns of attention that preceded goal-based and location-based predictions.

### Goal-based predictions

**Figure 2:**
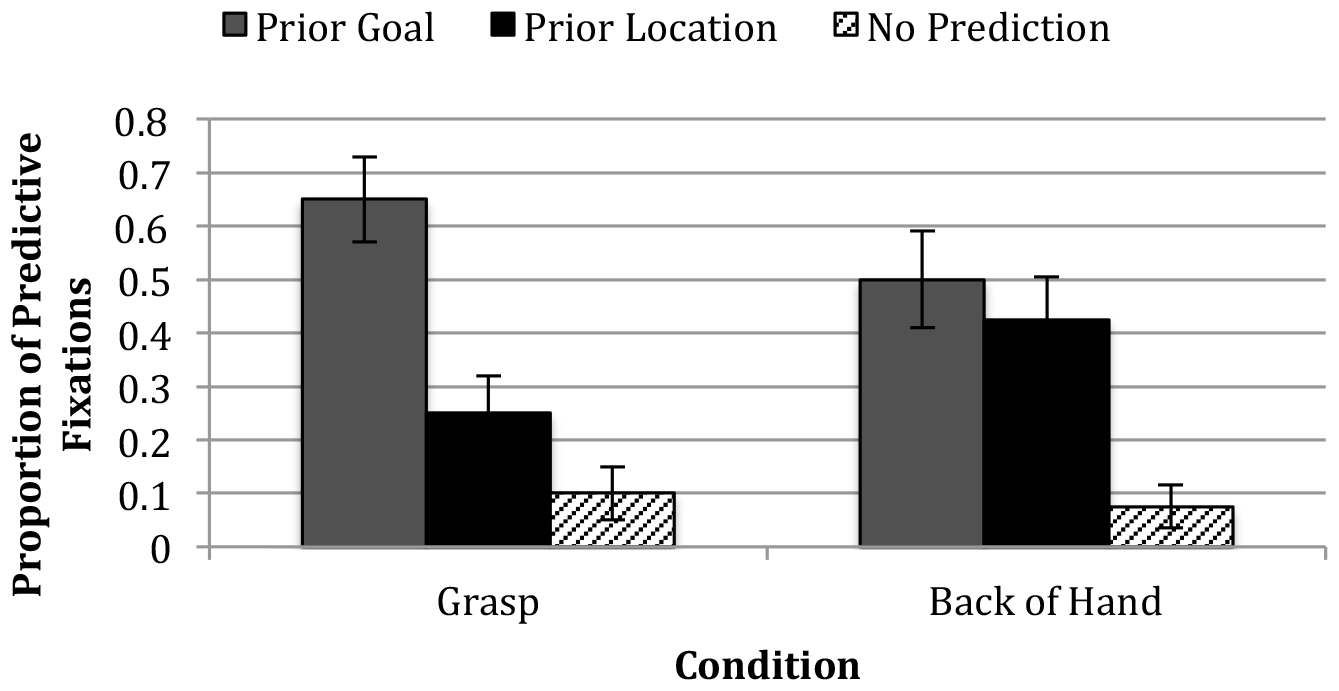
Results: Proportion of Predictive Fixations By Condition. Proportion of predictive fixations to either the prior goal or prior location object, or no prediction across the two test trials.

Figure 2 presents the proportion of test trials in which infants launched predictive fixations to either the prior goal or prior location object or made no prediction by condition. To focus our results on comparisons of prior goal versus prior location predictions, data from the No Prediction trials were removed from future analyses, which resulted in the exclusion of 4 individual test trials in the Grasp condition and 3 individual test trials in the Back-of-Hand condition. Infants' responses were averaged across trials, with each infant being assigned a final score of 0 (meaning only prior location predictions were generated), 0.5 (meaning 1 trial resulted in a prior goal prediction and 1 trial resulted in a prior location prediction), or 1 (meaning only prior goal predictions were generated). Initial analyses indicated no reliable effects of the infants' sex or age, as well as no reliable effects of which object was the target, the side on which the target sat, or which hand was used to perform the action. Therefore, subsequent analyses collapsed across these factors. The first analyses examined infants' predictive fixations to either the prior goal or prior location object during the test trials for the two conditions. Planned comparisons against chance (.50) revealed that infants in the Grasp condition launched predictive fixations systematically to the prior goal (*M* = .75, *SD* = .38, *t*(19) = 2.94, *p* = .01), whereas infants in the Back-of-Hand condition responded at chance levels (*M* = .55, *SD* = .36, *t*(19) = .62, *p* = .54). Infants' patterns of responding were similar across the two test trials. Given that the actor in the test trials never contacted the objects (as her hand paused centered between the two objects), these results show that infants generated goal-based predictions prior to exposure to information about the trajectory of the agent's movements and they attended to the intentionality of the action when generating these predictions. Consistent with results from passive habituation studies (e.g., [6]), infants in the Grasp condition produced more goal predictions than those in the Back-of-Hand condition, *t*(38) = 1.71, *p* = .04 (one-tailed).

Viewed nonparametrically, in the Grasp condition, 3 infants generated only location-based predictions, 4 infants generated both location- and goal-based predictions, and 13 infants generated only goal-based predictions (binomial *p* = .02). In the Back-of-Hand condition, 4 infants generated only location-based predictions, 10 infants generated both location- and goal-based predictions, and 6 infants generated only goal-based predictions (binomial *p* = .75). These patterns support the conclusion that infants in the Back-of-Hand condition generated goal-based predictions at chance levels, whereas those in the Grasp condition were above chance.

**Figure 3:**
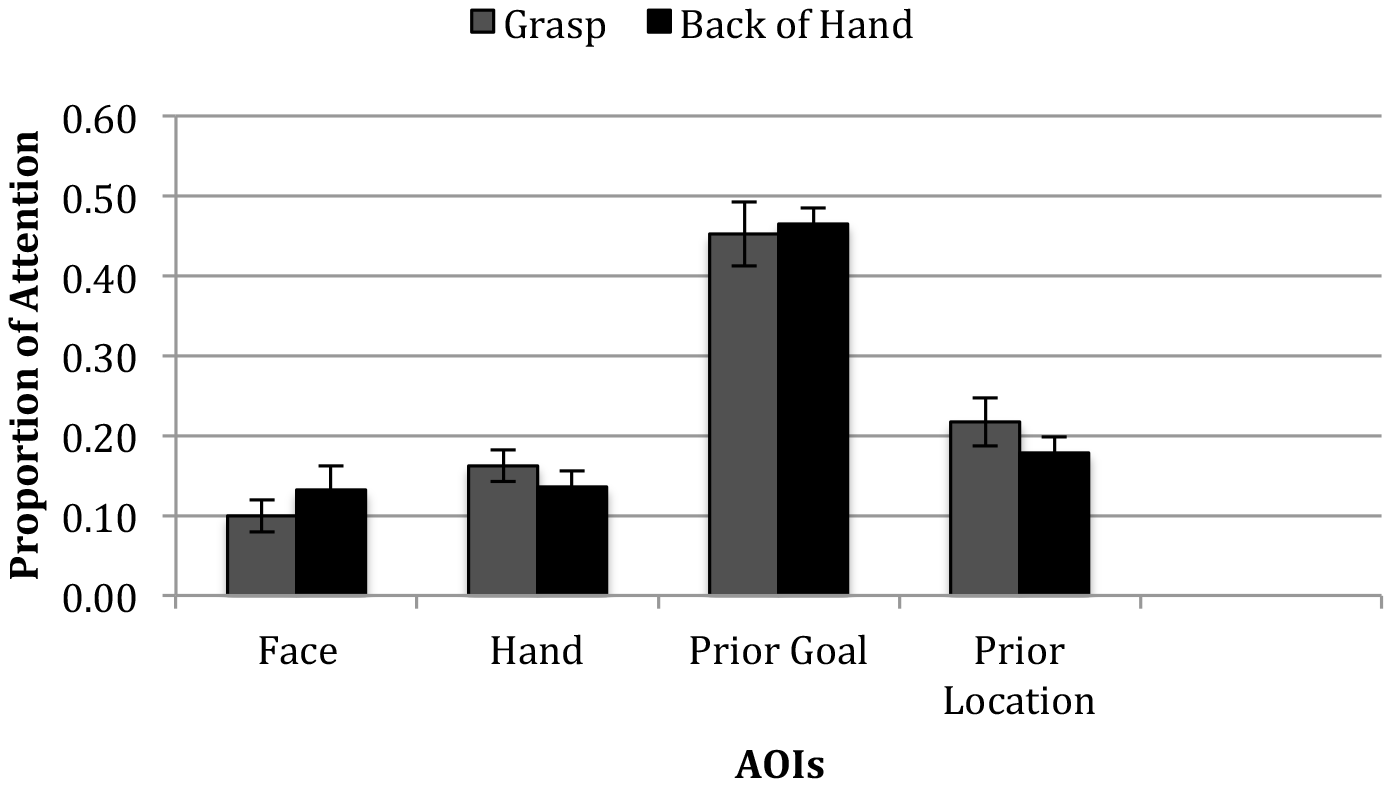
Results: Proportion of Visual Attention to the Familiarization Trial By Condition. Proportion of attention distributed across the four relevant action AOIs (i.e., Face, Hand, Prior Goal and Prior Location objects) during the single familiarization trial by condition.

Areas of interest (AOIs) were analyzed to evaluate whether infants' differential responses on test trials in the two conditions were driven by low-level differences in how their attention was entrained during familiarization. Infants' proportions of attention to the Face, Hand, Prior Goal, and Prior Location AOIs were calculated by dividing their attention to each relevant AOI by their total attention to the whole screen AOI for the familiarization trial. As shown in Figure 3, attention did not differ across the four AOIs during the familiarization trial across conditions. Additionally, there was no difference in infants' overall attention to the 7.5-sec familiarization event between the Grasp (*M* = 6.31-sec, *SD* = 1.37) and Back-of-Hand (*M* = 6.56-sec, *SD* = 1.17) conditions, *t*(38) = .62, *p* = .54. A regression examining whether infants' attention to the relevant AOIs during the familiarization trial predicted their scores for the test trials yielded no reliable effects (Face: *B* = .15; *t*(39) = .42, *p* = .68; Hand: *B* = .09; *t*(39) = 0.35, *p* = .73; Prior Goal: *B* = .09; *t*(39) = .21 *p* = .84; Prior Location: *B* = .30; *t*(39) = .84, *p* = .41). Thus, the differences in infants' predictive responses on test trials could not have derived from low-level differences in how their attention was recruited during familiarization trials. Additionally, the test trial stimuli were identical for the two conditions, so low-level properties of these events could not have driven infants' differential predictions in the two conditions. No difference was evident regarding infants' attention to the whole screen AOIs across the two test trials (Grasp: *M* = 5.82-sec, *SD* = 1.63; Back-of-Hand: *M* = 5.43-sec, *SD* = 1.35, *t*(38) = .81, *p* = .42). Thus, infants in the two conditions were equally attentive to the test events.

These findings indicate that, consistent with prior findings, infants systematically generated goal-based predictions when viewing well-formed grasping actions [17] and they provide novel evidence that infants differentiated between well-formed and ambiguous actions when doing so.

### Prediction latencies

**Figure 4:**
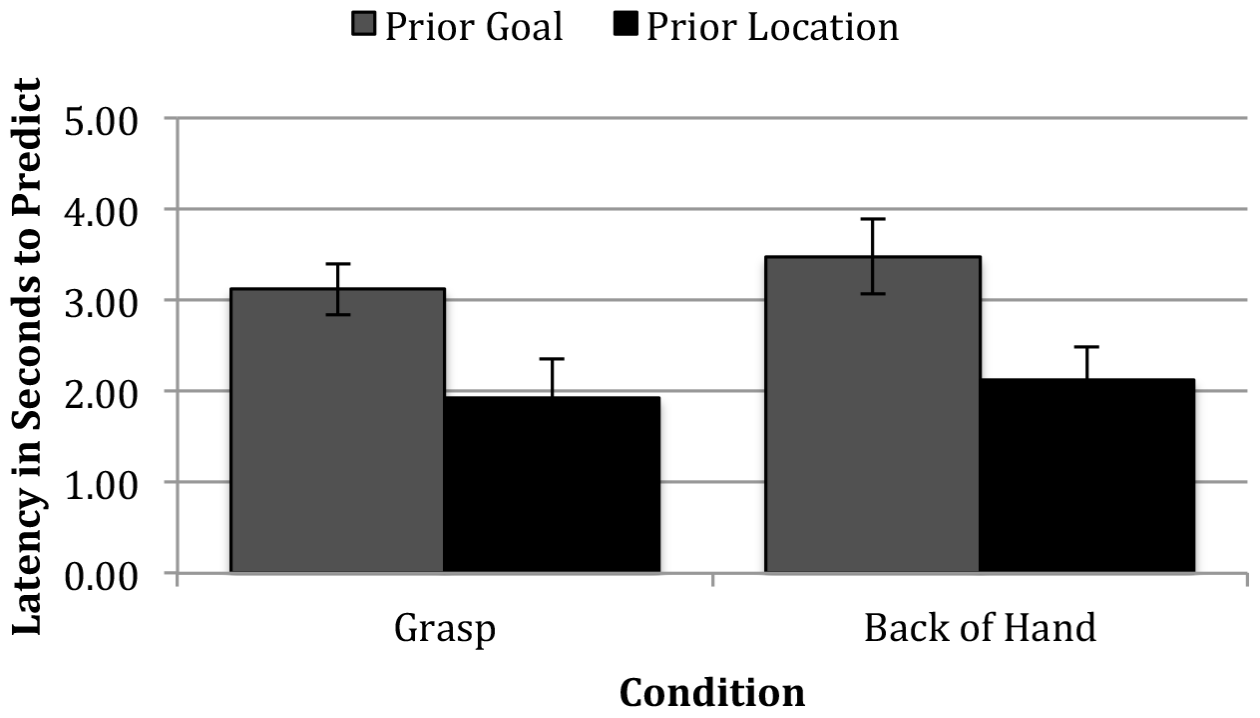
Average Latencies to Generate Predictive Fixations By Condition. Average latency in seconds to produce a predictive fixation to either the Prior Goal or Prior Location object across conditions.

Next, we evaluated the hypothesis that recruiting goal information requires cognitive resources that result in longer latencies when generating goal-based predictions than location-based predictions. We first considered this at the level of the individual trial. Latencies outside of two standard deviations from the mean for each condition were removed (2 trials in the Grasp condition and 1 trial in the Back-of-Hand condition). For the Grasp condition, the latencies from 25 trials were included as goal predictions, and the latencies from 9 trials were included as location predictions. For the Back-of-Hand condition, the latencies from 20 trials were included as goal predictions, and the latencies from 16 trials were included as location predictions. As described in the Data Reduction section, latencies were calculated from the start of the trial to the time point that infants fixated to an object AOI after the Hand AOI. Figure 4 presents the means and standard errors by condition. A binomial mixed-effect regression with infants' visual prediction responses (Prior Goal vs. Prior Location), condition (Grasp vs. Back-of-Hand), and test trial (test trial 1 vs. 2) as fixed effects, and participant as a random effect revealed that the only significant predictor of infants' latency to generate a prediction was infants' visual prediction response (response: *B* = −1.24; *t*(65) = −3.17, *p* = .002; condition: *B* = −0.26; *t*(36) = −0.66, *p* = .51; test trial: *B* = 0.06; *t*(34) = 0.18, *p* = .89). When infants predicted that the actor would continue to act upon the goal object, they took longer to produce predictive fixations, regardless of condition, than when they produced simpler location-based predictions.

We next evaluated the relation between prediction latency and prediction type at the participant level by conducting a correlational analysis across conditions to examine whether the infant's average prediction latency was related to his or her proportion of goal-based predictions. Results revealed a significant correlation such that longer average latencies were associated with higher goal-based prediction scores (*r*(40) = .43, *p* = .005). These results are consistent with the conclusion that recruiting goal information during action prediction is cognitively challenging.

### Attention prior to goal-based and location-based predictions

In the final set of analyses, we considered how infants allocated attention prior to generating goal-based versus location-based predictions in order to gain insight into the factors that may have led to the latency differences for these two kinds of predictions. Given that the latency differences indicate that infants attend more to the scene when producing goal-based predictions, it is of interest to determine whether there are more fine-grained differences in attention prior to generating a predictive fixation. Because infants demonstrated similar latency patterns in the Grasp and Back-of-Hand conditions, we combined the data across these conditions for these analyses. First, we calculated infants' proportions of attention to the 4 relevant AOIs (i.e., Face, Hand, Prior Goal, and Prior Location) prior to their generating a predictive fixation. These values were then averaged across test trials according to AOI category such that each infant received a single score for each AOI category. A regression was conducted to determine whether infants' attention to the AOIs predicted their scores for the test trials, i.e., was there a particular location, for example the actor's face or hand, that differentially drew attention prior to goal-based predictions. None of the factors significantly predicted infants' predictive gaze fixations to the goal during the test trials (Face: *B* = .04; *t*(39) = .24, *p* = .81; Hand: *B* = −0.16; *t*(39) = −0.88, *p* = .39; Prior Goal: *B* = −.19; *t*(34) = −1.06, *p* = .30; Prior Location: *B* = .22; *t*(34) = 1.20, *p* = .24). Thus, although overall latency to predict was related to infants' tendency to generate goal- versus location-based predictions, specific patterns of attention did not differ prior to the two kinds of predictions.

Another possibility is that the longer latencies for goal-based predictions reflected differences in the extent to which infants monitored each of the relevant regions in each event. In a second analysis, we evaluated infants' sampling of information from each AOI by assessing whether infants directed their gaze to each of the 4 critical regions (Face, Hand, Prior Goal, Prior Location) prior to generating a predictive look (all infants were required to look at the Hand AOI prior to producing a fixation; the predictive fixation, i.e., Prior Goal or Prior Location AOI, was not included in this analysis). A score of 0–4 was assigned for each test trial and then averaged across test trials such that each infant received a single sampling score. Infants' sampling scores were not correlated with their predictive fixation scores, *r*(40) = .18, *p* = .26. Therefore, infants' predictive fixations were not related to differential sampling of the scene prior to generating a prediction.

These findings reveal similar patterns of attention prior to generating goal-based and location-based predictions. It may be that heightened attention to the scene in general rather than specific patterns of attention was the critical indicator of successful judgments regarding an actor's future goal-directed behavior. Alternatively, making goal-based predictions may depend on the time required to compute the correct prediction, rather than on opportunities to attend to the scene per se. Further research is needed to address this issue. These findings and the prediction latency differences indicate that rushing to judgment is an unsuccessful strategy for infants at this age.

## General Discussion

Social competence requires the ability to assess others' intentions and to use this information to generate rapid predictions about their next actions. In the current study, 15-month-old infants showed this ability. Following a single familiarization trial, infants launched predictive eye movements to the goal of an agent when that agent had previously displayed the goal-directed action of grasping, providing support to the hypothesis that they were able to rapidly recruit their knowledge of her goal to predict her future behavior. This result adds to growing evidence that infants can generate rapid goal-based action predictions [17].

Our results also indicate that infants distinguish among different kinds of human movements when generating action predictions. When actions were ambiguous, as in the Back-of-Hand condition, infants did not generate systematic goal predictions, despite the fact that the timing and spatial characteristics of the actions were matched to the actions in the Grasp condition. Indeed, infants showed equivalent patterns of attention to the familiarization events across conditions, yet generated differential predictions in the subsequent test trials. This result indicates that infants engaged in a fine-grained analysis of the actions, rapidly, after viewing only a single familiarization trial.

One possibility is that infants were able to rapidly access their understanding of the grasping action to generate goal-based predictions because this action is more frequently experienced in their environment compared to the back-of-hand action. Evidence from habituation experiments suggests that infants may recruit contextual information (such as seeing an action repeatedly result in a specific outcome) to determine whether an ambiguous action is goal-directed (e.g., [20]). Future research should examine under which conditions infants see ambiguous gestures as meaningful and whether social information, perhaps over repeated trials or longer intervals, aids in their determinations.

Supporting the hypothesis that recruiting goal analysis is cognitively demanding, we found differences in infants' latency to produce a prediction to the prior goal as compared to the prior location. Across both conditions, longer processing times resulted in goal-based predictions as opposed to simpler location-based predictions. Predictions that involve more than anticipation of a movement regularity take longer, which is reflected in the amount of time infants required to recruit their knowledge of others' goals and to then deploy that knowledge to predict the most likely future behavior of their social partner. Infants attended in similar ways to the events prior to generating goal-based and location-based predictions, suggesting that it was spending more time evaluating the overall scene that mattered or perhaps simply the time required to compute the goal-based prediction, rather than a particular pattern of attention. One possibility is that infants who took longer to survey the information provided in the four relevant action AOIs were more able to update their representation of the event during test trials. Upon detecting that the event had changed, infants could recruit their updated knowledge to accurately predict that the agent would continue to act upon the prior goal object.

The difference in prediction latencies highlights a distinction between the cognitive demands in our task compared to those in movement regularity paradigms, as infants who relied on a simple motor perseverance response would have produced location-based predictions in our paradigm. Our stricter definition of action anticipation as well as the use of an incomplete action during the test trials allows for a more in-depth understanding of infants' ability to anticipate the future behavior of others and the time course for the production of these anticipations.

Moreover, the current study provides further evidence that infants are able to generate goal-based predictions (e.g., [17]) and contrasts with results from studies using unusual, animated characters in complicated scenes to assess action predictions (i.e., [18], [19]). The possibility remains open that infants' failure to generate goal-based predictions in these previous studies derived from timing limitations. Perhaps infants needed more time than was available in those experiments to analyze the unusual events and then generate cognitively informed predictions. Thus, prior findings together with the results of the current study suggest that providing rich information regarding the animacy of the agent, a familiar action context, and a complex social environment may support infants' ability to think quickly about goal-based actions.

Our findings may also help to explain an apparent contradiction in the literature on infant social understanding. Converging evidence from passive experimental methods indicates that preverbal infants have an understanding of others' intentions and goals early in the first postnatal year (e.g., [1], [4], [5], [21]–[23]). However, infants do not appear as sophisticated in their knowledge of others during naturalistic interactions until later in development. During infant-controlled looking time procedures, infants are in control of the amount of time provided to encode the scene, whereas real-time social interactions require contingent exchanges between social partners that are performed with fine-grained temporal precision. Young infants may not evidence an understanding of others' goals during these interactions because they are unable to rapidly recruit their knowledge to continue the social exchange. It is possible, therefore, that the developments in social interactive competence during the second year of life may reflect increases in the speed with which infants can employ their knowledge during fast-paced social interactions. Further research is needed to evaluate this possibility.

These open questions aside, the current findings highlight the importance of not only being a “smart” social partner but the need to be a “fast” social thinker as well. Infants in their first years of life learn a great deal from their social partners, and this is essential for acquiring language and cultural norms. To effectively learn from others, infants must attend to their intentions and be able to quickly use this social information to formulate responses that will foster interactive exchanges. A failure to respond quickly on the infant's part could lead to the end of the social interaction and the learning opportunities it provides. Most current research on early social cognition has focused on *what* infants know about others at different points in development. Our findings highlight the need to also understand the factors that allow infants to *deploy* this knowledge in real time.
